# An effective detection approach for phishing websites using URL and HTML features

**DOI:** 10.1038/s41598-022-10841-5

**Published:** 2022-05-25

**Authors:** Ali Aljofey, Qingshan Jiang, Abdur Rasool, Hui Chen, Wenyin Liu, Qiang Qu, Yang Wang

**Affiliations:** 1 Shenzhen Key Laboratory for High Performance Data Mining, Shenzhen Institute of Advanced Technology, Chinese Academy of Sciences, Shenzhen, 518055 China; 2 Shenzhen College of Advanced Technology, University of Chinese Academy of Sciences, Beijing, 100049 China; 3 Department of Computer Science, Guangdong University of Technology, Guangzhou, China; 4 Cloud Computing Center, Shenzhen Institute of Advanced Technology, Chinese Academy of Sciences, Shenzhen, 518055 China

**Keywords:** Computer science, Information technology, Scientific data

## Abstract

Today's growing phishing websites pose significant threats due to their extremely undetectable risk. They anticipate internet users to mistake them as genuine ones in order to reveal user information and privacy, such as login ids, pass-words, credit card numbers, etc. without notice. This paper proposes a new approach to solve the anti-phishing problem. The new features of this approach can be represented by URL character sequence without phishing prior knowledge, various hyperlink information, and textual content of the webpage, which are combined and fed to train the XGBoost classifier. One of the major contributions of this paper is the selection of different new features, which are capable enough to detect 0-h attacks, and these features do not depend on any third-party services. In particular, we extract character level Term Frequency-Inverse Document Frequency (TF-IDF) features from noisy parts of HTML and plaintext of the given webpage. Moreover, our proposed hyperlink features determine the relationship between the content and the URL of a webpage. Due to the absence of publicly available large phishing data sets, we needed to create our own data set with 60,252 webpages to validate the proposed solution. This data contains 32,972 benign webpages and 27,280 phishing webpages. For evaluations, the performance of each category of the proposed feature set is evaluated, and various classification algorithms are employed. From the empirical results, it was observed that the proposed individual features are valuable for phishing detection. However, the integration of all the features improves the detection of phishing sites with significant accuracy. The proposed approach achieved an accuracy of 96.76% with only 1.39% false-positive rate on our dataset, and an accuracy of 98.48% with 2.09% false-positive rate on benchmark dataset, which outperforms the existing baseline approaches.

## Introduction

Phishing offenses are increasing, resulting in billions of dollars in loss^1^. In these attacks, users enter their critical (i.e., credit card details, passwords, etc.) to the forged website which appears to be legitimate. The Software-as-a-Service (SaaS) and webmail sites are the most common targets of phishing^2^. The phisher makes websites that look very similar to the benign websites. The phishing website link is then sent to millions of internet users via emails and other communication media. These types of cyber-attacks are usually activated by emails, instant messages, or phone calls^3^. The aim of the phishing attack is not only to steal the victims' personality, but it can also be performed to spread other types of malware such as ransomware, to exploit approach weaknesses, or to receive monetary profits^4^. According to the Anti-Phishing Working Group (APWG) report in the 3rd Quarter of 2020, the number of phishing attacks has grown since March, and 28,093 unique phishing sites have been detected between July to September^2^. The average amount demanded during wire transfer Business E-mail Compromise (BEC) attacks was $48,000 in the third quarter, down from $80,000 in the second quarter and $54,000 in the first.

Detecting and preventing phishing offenses is a significant challenge for researchers due to the way phishers carry out the attack to bypass the existing anti-phishing techniques. Moreover, the phisher can even target some educated and experienced users by using new phishing scams. Thus, software-based phishing detection techniques are preferred for fighting against the phishing attack. Mostly available methods for detecting phishing attacks are blacklists/whitelists^5^, natural language processing^6^, visual similarity^7^, rules^8^, machine learning techniques ^9,10^, etc. Techniques based on blacklists/whitelists fail to detect unlisted phishing sites (i.e. 0-h attacks) as well as these methods fail when blacklisted URL is encountered with minor changes. In the machine learning based techniques, a classification model is trained using various heuristic features (i.e., URL, webpage content, website traffic, search engine, WHOIS record, and Page Rank) in order to improve detection efficiency. However, these heuristic features are not warranted to present in all phishing websites and might also present in the benign websites, which may cause a classification error. Moreover, some of the heuristic features are hard to access and third-party dependent. Some third-party services (i.e., page rank, search engine indexing, WHOIS etc.) may not be sufficient to identify phishing websites that are hosted on hacked servers and these websites are inaccurately identified as benign websites because they are contained in search results. Websites hosted on compromised servers are usually more than a day old unlike other phishing websites which only take a few hours. Also, these services inaccurately identify the new benign website as a phishing site due to the lack of domain age. The visual similarity-based heuristic techniques compare the new website with the pre-stored signature of the website. The website’s visual signature includes screenshots, font styles, images, page layouts, logos, etc. Thus, these techniques cannot identify the fresh phishing websites and generate a high false-negative rate (phishing to benign). The URL based technique does not consider the HTML of the webpage and may misjudge some of the malicious websites hosted on free or compromised servers. Many existing approaches^11–13^ extract hand-crafted URL based features, e.g., number of dots, presence of special “@”, “#”, “–” symbol, URL length, brand names in URL, position of Top-Level domain, check hostname for IP address, presence of multiple TLDs, etc. However, there are still hurdles to extracting manual URL features due to the fact that human effort requires time and extra maintenance labor costs. Detecting and preventing phishing offense is a major defiance for researchers because the scammer carries out these offenses in a way that can avoid current anti-phishing methods. Hence, the use of hybrid methods rather than a single approach is highly recommended by the networks security manager.

This paper provides an efficient solution for phishing detection that extracts the features from website's URL and HTML source code. Specifically, we proposed a hybrid feature set including URL character sequence features without expert’s knowledge, various hyperlink information, plaintext and noisy HTML data-based features within the HTML source code. These features are then used to create feature vector required for training the proposed approach by XGBoost classifier. Extensive experiments show that the proposed anti-phishing approach has attained competitive performance on real dataset in terms of different evaluation statistics.

Our anti-phishing approach has been designed to meet the following requirements.

- High detection efficiency: To provide high detection efficiency, incorrect classification of benign sites as phishing (false-positive) should be minimal and correct classification of phishing sites (true-positive) should be high.
- Real-time detection: The prediction of the phishing detection approach must be provided before exposing the user's personal information on the phishing website.
- Target independent: Due to the features extracted from both URL and HTML the proposed approach can detect new phishing websites targeting any benign website (zero-day attack).
- Third-party independent: The feature set defined in our work are lightweight and client-side adaptable, which do not rely on third-party services such as blacklist/whitelist, Domain Name System (DNS) records, WHOIS record (domain age), search engine indexing, network traffic measures, etc. Though third-party services may raise the effectiveness of the detection approach, they might misclassify benign websites if a benign website is newly registered. Furthermore, the DNS database and domain age record may be poisoned and lead to false negative results (phishing to benign).
  Hence, a light-weight technique is needed for phishing websites detection adaptable at client side. The major contributions in this paper are itemized as follows.
- We propose a phishing detection approach, which extracts efficient features from the URL and HTML of the given webpage without relying on third-party services. Thus, it can be adaptable at the client side and specify better privacy.
- We proposed eight novel features including URL character sequence features (F1), textual content character level (F2), various hyperlink features (F3, F4, F5, F6, F7, and F14) along with seven existing features adopted from the literature.
- We conducted extensive experiments using various machine learning algorithms to measure the efficiency of the proposed features. Evaluation results manifest that the proposed approach precisely identifies the legitimate websites as it has a high true negative rate and very less false positive rate.
- We release a real phishing webpage detection dataset to be used by other researchers on this topic.

The rest of this paper is structured as follows: The "Related work" section first reviews the related works about phishing detection. Then the "Proposed approach" section presents an overview of our proposed solution and describes the proposed features set to train the machine learning algorithms. The "Experiments and result analysis” section introduces extensive experiments including the experimental dataset and results evaluations. Furthermore, the "Discussion and limitation" section contains a discussion and limitations of the proposed approach. Finally, the "Conclusion" section concludes the paper and discusses future work.

## Related work

This section provides an overview of the proposed phishing detection techniques in the literature. Phishing methods are divided into two categories; expanding the user awareness to distinguish the characteristics of phishing and benign webpages^14^, and using some extra software. Software-based techniques are further categorized into list-based detection, and machine learning-based detection. However, the problem of phishing is so sophisticated that there is no definitive solution to efficiently bypass all threats; thus, multiple techniques are often dedicated to restrain particular phishing offenses.

### List-based detection

List-based phishing detection methods use either whitelist or blacklist-based technique. A blacklist contains a list of suspicious domains, URLs, and IP addresses, which are used to validate if a URL is fraudulent. Simultaneously, the whitelist is a list of legitimate domains, URLs, and IP addresses used to validate a suspected URL. Wang et al.^15^, Jain and Gupta^5^ and Han et al.^16^ use white list-based method for the detection of suspected URL. Blacklist-based methods are widely used in openly available anti-phishing toolbars, such as Google safe browsing, which maintains a blacklist of URLs and provides warnings to users once a URL is considered as phishing. Prakash et al.^17^ proposed a technique to predict phishing URLs called Phishnet. In this technique, phishing URLs are identified from the existing blacklisted URLs using the directory structure, equivalent IP address, and brand name. Felegyhazi et al.^18^ developed a method that compares the domain name and name server information of new suspicious URLs to the information of blacklisted URLs for the classification process. Sheng et al.^19^ demonstrated that a forged domain was added to the blacklist after a considerable amount of time, and approximately 50–80% of the forged domains were appended after the attack was carried out. Since thousands of deceptive websites are launched every day, the blacklist requires to be updated periodically from its source. Thus, machine learning-based detection techniques are more efficient in dealing with phishing offenses.

### Machine learning-based detection

Data mining techniques have provided outstanding performance in many applications, e.g., data security and privacy^20^, game theory^21^, blockchain systems^22^, healthcare^23^, etc. Due to the recent development of phishing detection methods, various machine learning-based techniques have also been employed^6,9,10,13^ to investigate the legality of websites. The effectiveness of these methods relies on feature collection, training data, and classification algorithm. The feature collection is extracted from different sources, e.g., URL, webpage content, third party services, etc. However, some of the heuristic features are hard to access and time-consuming, which makes some machine learning approaches demand high computations to extract these features.

Jain and Gupta^24^ proposed an anti-phishing approach that extracts the features from the URL and source code of the webpage and does not rely on any third-party services. Although the proposed approach attained high accuracy in detecting phishing webpages, it used a limited dataset (2141 phishing and 1918 legitimate webpages). The same authors^9^ present a phishing detection method that can identify phishing attacks by analyzing the hyperlinks extracted from the HTML of the webpage. The proposed method is a client-side and language-independent solution. However, it entirely depends on the HTML of the webpage and may incorrectly classify the phishing webpages if the attacker changes all webpage resource references (i.e., Javascript, CSS, images, etc.). Rao and Pais^25^ proposed a two-level anti-phishing technique called BlackPhish. At first level, a blacklist of signatures is created using visual similarity based features (i.e., file names, paths, and screenshots) rather than using blacklist of URLs. At second level, heuristic features are extracted from URL and HTML to identify the phishing websites which override the first level filter. In spite of that, the legitimate websites always undergo two-level filtering. In some researches^26^ authors used search engine-based mechanism to authenticate the webpage as first-level authentication. In the second level authentication, various hyperlinks within the HTML of the website are processed for the phishing websites detection. Although the use of search engine-based techniques increases the number of legitimate websites correctly identified as legitimate, it also increases the number of legitimate websites incorrectly identified as phishing when newly created authentic websites are not found in the top results of search engine. Search based approaches assume that genuine website appears in the top search results.

In a recent study, Rao et al.^27^ proposed a new phishing websites detection method with word embedding extracted from plain text and domain specific text of the html source code. They implemented different word embedding to evaluate their model using ensemble and multimodal techniques. However, the proposed method is entirely dependent on plain text and domain specific text, and may fail when the text is replaced with images. Some researchers have tried to identify phishing attacks by extracting different hyperlink relationships from webpages. Guo et al.^28^ proposed a phishing webpages detection approach which they called HinPhish. The approach establishes a heterogeneous information network (HIN) based on domain nodes and loading resources nodes and establishes three relationships between the four hyperlinks: external link, empty link, internal link and relative link. Then, they applied an authority ranking algorithm to calculate the effect of different relationships and obtain a quantitative score for each node.

In Sahingoz et al.^6^ work, the distributed representation of words is adopted within a specific URL, and then seven various machine learning classifiers are employed to identify whether a suspicious URL is a phishing website. Rao et al.^13^ proposed an anti-phishing technique called CatchPhish. They extracted hand-crafted and Term Frequency-Inverse Document Frequency (TF-IDF) features from URLs, then trained a classifier on the features using random forest algorithm. Although the above methods have shown satisfactory performance, they suffer from the following restrictions: (1) inability to handle unobserved characters because the URLs usually contain meaningless and unknown words that are not in the training set; (2) they do not consider the content of the website. Accordingly, some URLs, which are distinctive to others but imitate the legitimate sites, may not be identified based on URL string. As their work is only based on URL features, which is not enough to detect the phishing websites. However, we have provided an effective solution by proposing our approach to this domain by utilizing three different types of features to detect the phishing website more efficiently. Specifically, we proposed a hybrid feature set consisting of URL character sequence, various hyperlinks information, and textual content-based features.

Deep learning methods have been used for phishing detection e.g., Convolutional Neural Network (CNN), Deep Neural Network (DNN), Recurrent Neural Network (RNN), and Recurrent Convolutional Neural Networks (RCNN) due to the success of the Natural Language Processing (NLP) attained by these techniques. However, deep learning methods are not employed much in phishing detection due to the inclusive training time. Aljofey et al.^3^ proposed a phishing detection approach with a character level convolutional neural network based on URL. The proposed approach was compared by using various machine and deep learning algorithms, and different types of features such as TF-IDF characters, count vectors, and manually-crafted features. Le et al.^29^ provided a URLNet method to detect phishing webpage from URL. They extract character-level and word-level features from URL strings and employ CNN networks for training and testing. Chatterjee and Namin^30^ introduced a phishing detection technique based on deep reinforcement learning to identify phishing URLs. They used their model on a balanced, labeled dataset of benign and phishing URLs, extracting 14 hand-crafted features from the given URLs to train the proposed model. In recent studies, Xiao et al.^31^ proposed phishing website detection approach named CNN–MHSA. CNN network is applied to extract characters features from URLs. In the meanwhile, multi-head self-attention (MHSA) mechanism is employed to calculate the corresponding weights for the CNN learned features. Zheng et al.^32^ proposed a new Highway Deep Pyramid Neural Network (HDP-CNN) which is a deep convolutional network that integrates both character-level and word-level embedding representation to identify whether a given URL is phishing or legitimate. Albeit the above approaches have shown valuable performances, they might misclassify phishing websites hosted on compromised servers since the features are extracted only from the URL of the website.

The features extracted in some previous studies are based on manual work and require additional effort since these features need to be reset according to the dataset, which may lead to overfitting of anti-phishing solutions. We got the motivation from the above-mentioned studies and proposed our approach. In which, the current work extract character sequences feature from URL without manual intervention. Moreover, our approach employs noisy data of HTML, plaintext, and hyperlinks information of the website with the benefit of identifying new phishing websites. Table 1 presents the detailed comparison of existing machine learning based phishing detection approaches.

**Table 1:** Comparison of machine learning based phishing detection approaches.

Approach	Description	Dataset	Limitations
Jain and Gupta^24^	This approach filters phishing websites at client side based on handcrafted URL features, hyperlinks features, and identity keywords features using Random Forest	A private dataset of 2141 phishing webpages and 1918 benign webpages	It extracts manually designed URL features, which need human effort Identity features are language dependent where top key words are extracted from website
Jain and Gupta^9^	Proposed an anti-phishing approach using logistic regression, which relies on various hyperlink features extracted from the HTML content of webpage	A private dataset of 1428 phishing and 1116 benign webpages	Limited dataset The feature set completely depends on the webpage content which fails when content is replaced by Images
Rao and Pais^25^	Authors developed a two level filtering technique to detect phishing sites using enhanced blacklist and heuristic features	A public dataset of 5438 benign and 4097 Phishing webpages	The benign sites always go through two level filtering
Jain and Gupta^26^	An approach to classify the websites based on two level authentications: search engine and hyperlink information	A private dataset of 2000 benign and 2000 phishing webpages	Fails at first level when newly constructed benign sites do not appear in top search results Fails when content of webpage is replaced by an image
Sahingoz et al.^6^	Use NLP based features, word vectors, and hybrid features, and then seven different machine learning algorithms are used to classify the URLs	A public dataset of 36,400 benign URLs and 37,175 phishing URLs	Inability to handle unseen characters in URLs The method may fail to detect the shorter URLs
Rao et al.^13^	This technique proposes manually crafted URL features and TF-IDF based features and with the use of these features classifies the URLs by using random forest classifier	A public dataset of 85,409 benign URLs and 40,668 phishing URLs	Extracts hand-crafted URL features, which need human effort and additional maintenance labor costs The model may fails when phishing sites hosted on free or compromised hosting servers
Aljofey et al.^3^	A fast deep learning model based on the URL, which uses character-level CNN, is proposed for phishing detection	A private dataset of 157,626 benign URLs and 161,016 phishing URLs	It completely depends on the URL of the website It does not interest if the URL of the website is alive or if there is an error
Le et al.^29^	This technique applies CNN networks to both characters and words of the URL string for malicious URL detection	A private dataset of 4,683,425 benign URLs and 9,366,850 malicious URLs	Since the deep learning model implemented with both word-level and character-level embedding, it requires sufficient memory
Xiao et al.^31^	Proposed a technique named CNN–MHSA, which combines convolutional neural network (CNN) and multi-head self-attention (MHSA) mechanism together to learn features in URLs and detect phishing	A private dataset where 45,000 are benign and 43,984 are phishing	The URL length parameter may affect the robustness of the model
Zheng et al.^32^	Proposed a new Highway Hierarchical Neural Network (HDP-CNN) to detect phishing URLs. This method uses word-level embedding along with character-level embedding to exhibit better performance	A private dataset contains 344,794 benign URLs and 71,556 phishing URLs	The problem of severe data imbalance is probably causing the model to overfit on large datasets
Rao et al.^27^	A machine leaning technique that uses word embedding algorithms to generate a feature vector using plain text and domain text extracted from the webpage content	A public dataset consists of 5438 phishing websites and 5076 benign websites with their URLs	The technique is language dependent It fails when content of webpage is replaced by an image
Guo et al.^28^	A phishing detection approach that creates heterogeneous information networks based on domain nodes, page resource nodes, and relationships between hyperlinks	A public dataset contains 29,496 phish samples and 30,649 benign samples	The approach may exhibit poor performance when the webpage contains a few number of hyperlinks
Proposed approach	A machine learning approach that consists of a hybrid feature set including URL character sequence, different hyperlink features, and TF-IDF character level features from the plaintext and noisy part of the given webpage's HTML	A public data set consisting of 27,280 phishing URLs with HTML codes and 32,972 benign pages	The plain text-based feature of a webpage is language-based Need for accessing the HTML source code of webpage

### Proposed approach

Our approach extracts and analyzes different features of suspected webpages for effective identification of large-scale phishing offenses. The main contribution of this paper is the combined uses of these feature set. For improving the detection accuracy of phishing webpages, we have proposed eight new features. Our proposed features determine the relationship between the URL of the webpage and the webpage content.

### System architecture

**Figure 1:**
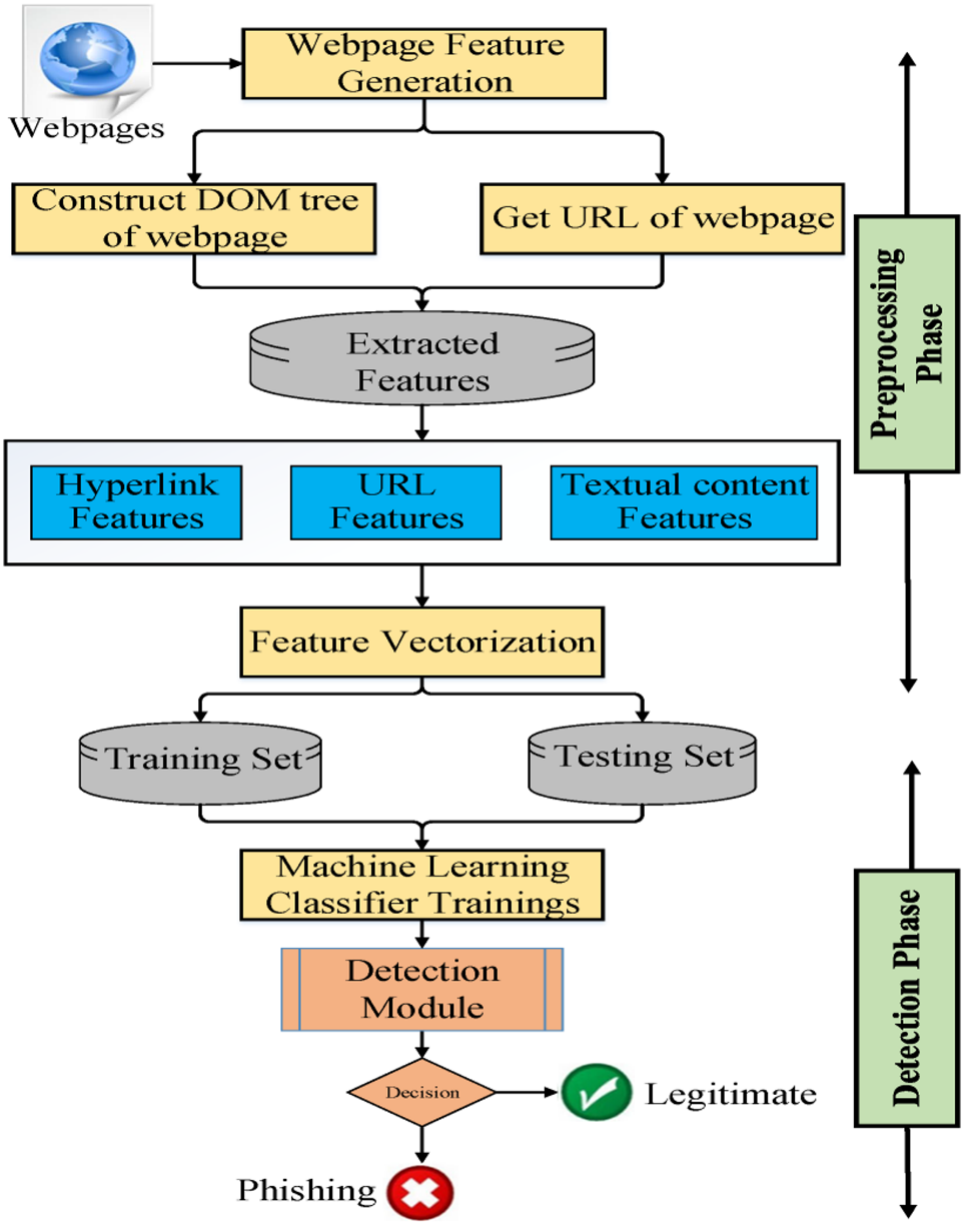
General architecture of the proposed approach.

The overall architecture of the proposed approach is divided into three phases. In the first phase, all the essential features are extracted and HTML source code will be crawled. The second phase applies feature vectorization to generate a particular feature vector for each webpage. The third phase identifies if the given webpage is phishing. Figure 1 shows the system structure of the proposed approach. Details of each phase are described as follows.

#### Feature generation

The features are generated in this component. Our features are based on the URL and HTML source code of the webpage. A Document Object Model (DOM) tree of the webpage is used to extract the hyperlink and textual content features using a web crawler automatically. The features of our approach are categorized into four groups as depicted in Table 2. In particular, features F1–F7, and F14 are new and proposed by us; Features F8–F13, and F15 are taken from other approaches^9,11,12,24,33^ but we adjusted them for better results. Moreover, the observational method and strategy regarding the interpretation of these features are applied differently in our approach. A detailed explanation of the proposed features is provided in the feature extraction section of this paper.

**Table 2:** Features used in the proposed approach.

Category	No	Name
URL based features	F1	Character sequences vectors
Textual content features	F2	TF-IDF vector N-gram chars
Hyperlink information	F3, F4, F5, F6, F7, F8, F9, F10, F11, F12, and F13	Script_files, CSS_files, img_files, a_files, a_Null_hyperlinks, Null_hyperlinks, Total_hyperlinks, Internal_hyperlinks, External_hyperlinks, External/Internal_hyperlinks, and Error_hyperlinks
Login form information	F14 and F15	Total_forms and Suspicious_form

#### Feature vectorization

After the features are extracted, we apply feature vectorization to generate a particular feature vector for each webpage to create a labeled dataset. We integrate URL character sequences features with textual content TF-IDF features and hyperlink information features to create feature vector required for training the proposed approach. The hyperlink features combination outputs 13-dimensional feature vector as $F_{H} = \left\langle {f_{3} ,f_{4} ,f_{5} , \ldots ,f_{{15}} } \right\rangle$, and the URL character sequence features combination outputs 200-dimensional feature vector as $F_{U} = \left\langle {c_{1} ,c_{2} ,c_{3} , \ldots ,c_{{200}} } \right\rangle$, we set a fixed URL length to 200. If the URL length is greater than 200, the additional part will be ignored. Otherwise, we put a 0 in the remainder of the URL string. The setting of this value depends on the distribution of URL lengths within our dataset. We have noticed that most of the URL lengths are less than 200 which means that when a vector is long, it may contain useless information, in contrast when the feature vector is too short, it may contain insufficient features. TF-IDF character level combination outputs $D$-dimensional feature vector as $F_{T} = \left\langle {t_{1} ,t_{2} ,t_{3} , \ldots ,t_{D} } \right\rangle$ where $D$ is the size of dictionary computed from the textual content corpus. It is observed from the experimental analysis that the size of dictionary $D$ = 20,332 and the size increases with an increase in number of corpus. The above three feature vectors are combined to generate final feature vector $F_{V} = F_{T} \cup F_{U} \cup F_{H} = \left\langle {t_{1} ,t_{2} , \ldots ,t_{D} ,c_{1} ,c_{2} \ldots ,c_{{200}} ,f_{3} ,f_{4} ,f_{5} , \ldots ,f_{{15}} } \right\rangle$ that is fed as input to machine learning algorithms to classify the website.

#### Detection module

**Figure 2:**
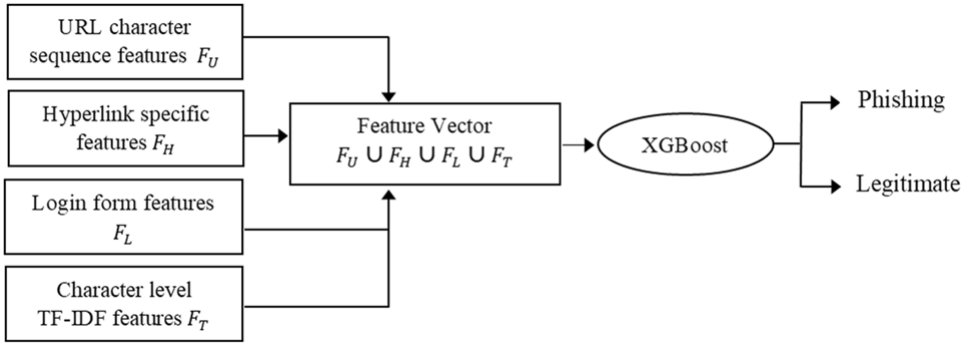
Phishing detection algorithm.

The Detection phase includes building a strong classifier by using the boosting method, XGBoost classifier. Boosting integrates many weak and relatively accurate classifiers to build a strong and therefore robust classifier for detecting phishing offences. Boosting also helps to combine diverse features resulting in improved classification performance^34^. Here, XGBoost classifier is employed on integrated feature sets of URL character sequence ${F}_{U}$, various hyperlinks information ${F}_{H}$, login form features ${F}_{L}$, and textual content-based features ${F}_{T}$ to build a strong classifier for phishing detection. In the training phase, XGBoost classifier is trained using the feature vector $({F}_{U}\cup {F}_{H} \cup {F}_{L} \cup {F}_{T})$ collected from each record in the training dataset. At the testing phase, the classifier detects whether a particular website is a malicious website or not. The detailed description is shown in Fig. 2.

### Features extraction

Due to the limited search engine and third-party methods discussed in the literature, we extract the particular features from the client side in our approach. We have introduced eleven hyperlink features (F3–F13), two login form features (F14 and F15), character level TF-IDF features (F2), and URL character sequence features (F1). All these features are discussed in the following subsections.

#### URL character sequence features (F1)

The URL stands for Uniform Resource Locator. It is used for providing the location of the resources on the web such as images, files, hypertext, video, etc. URL. Each URL starts with a protocol (http, https, and ftp) used to access the resource requested. In this part, we extract character sequence features from URL. We employ the method used in^35^ to process the URL at the character level. More information is contained at the character level. Phishers also imitate the URLs of legitimate websites by changing many unnoticeable characters, e.g., “www.icbc.com” as “www.1cbc.com”. Character level URL processing is a solution to the out of vocabulary problem. Character level sequences identify substantial information from specific groups of characters that appear together which could be a symptom of phishing. In general, a URL is a string of characters or words where some words have little semantic meanings. Character sequences help find this sensitive information and improve the efficiency of phishing URL detection. During the learning task, machine learning techniques can be applied directly using the extracted character sequence features without the expert intervention. The main processes of character sequences generating include: preparing the character vocabulary, creating a tokenizer object using Keras preprocessing package (https://Keras.io) to process URLs in char level and add a “UNK” token to the vocabulary after the max value of chars dictionary, transforming text of URLs to sequence of tokens, and padding the sequence of URLs to ensure equal length vectors. The description of URL features extraction is shown in Algorithm 1.
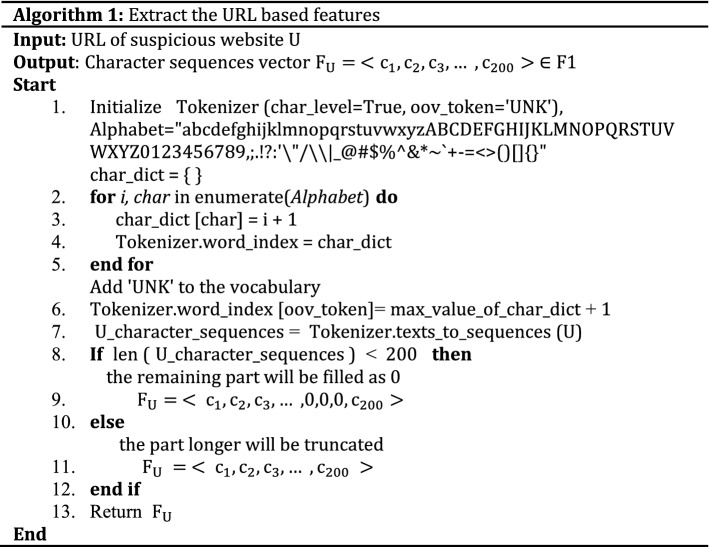


### HTML features

The webpage source code is the programming behind any webpage, or software. In case of websites, this code can be viewed by anyone using various tools, even in the web browser itself. In this section, we extract the textual and hyperlink features existing in the HTML source code of the webpage.

#### Textual content-based features (F2)

**Figure 3:**
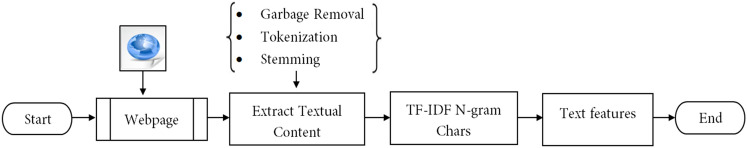
The process of generating text features.

TF-IDF stands for Term Frequency-Inverse Document Frequency. TF-IDF weight is a statistical measure that tells us the importance of a term in a corpus of documents^36^. TF-IDF vectors can be created at various levels of input tokens (words, characters, n-grams) ^37^. It is observed that TF-IDF technique has been implemented in many approaches to catch phish of webpages by inspecting URLs ^13^, obtain the indirect associated links^38^, target website^11^, and validity of suspected website ^39^. In spite of TF-IDF technique extracts outstanding keywords from the text content of the webpage, it has some limitations. One of the limitations is that TF-IDF technique fails when the extracted keywords are meaningless, misspelled, skipped or replaced with images. Since plaintext and noisy data (i.e., attribute values for div, h1, h2, body and form tags) are extracted in our approach from the given webpage using BeautifulSoup parser, TF-IDF character level technique is applied with max features as 25,000. To obtain valid textual information, extra portions (i.e., JavaScript code, CSS code, punctuation symbols, and numbers) of the webpage are removed through regular expressions, including Natural Language Processing packages (http://www.nltk.org/nltk_data/) such as sentence segmentation, word tokenization, text lemmatization and stemming as shown in Fig. 3.

Phishers usually mimic the textual content of the target website to trick the user. Moreover, phishers may mistake or override some texts (i.e., title, copyright, metadata, etc.) and tags in phishing webpages to bypass revealing the actual identification of the webpage. However, tag attributes stay the same to preserve the visual similarity between phishing and targeted site using the same style and theme as that of the benign webpage. Therefore, it is needful to extract the text features (plaintext and noisy part of HTML) of the webpage. The basic of this step is to extract the vectored representation of the text and the effective webpage content. A TF-IDF object is employed to vectorize text of the webpage. The detailed process of the text vector generation algorithm as follows.
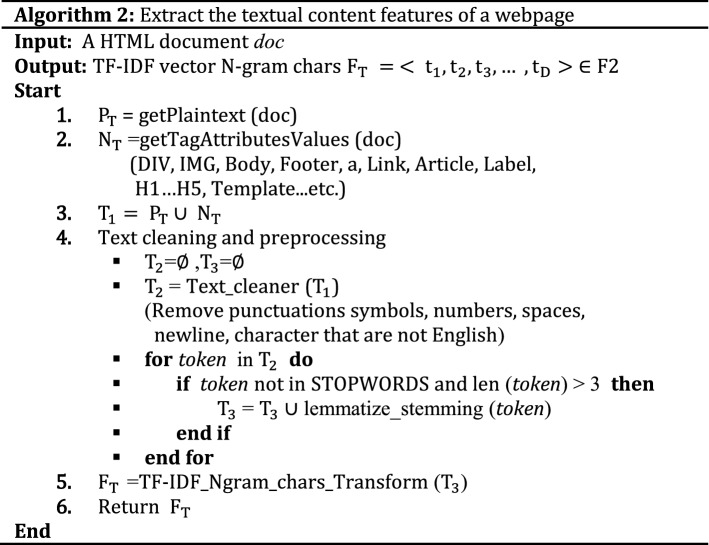


#### Script, CSS, img, and anchor files (F3, F4, F5, and F6)

External JavaScript or external Cascading Style Sheets (CSS) files are separate files that can be accessed by creating a link within the head section of a webpage. JavaScript, CSS, images, etc. files may contain malicious code while loading a webpage or clicking on a specific link. Moreover, phishing websites have fragile and unprofessional content as the number of hyperlinks referring to a different domain name increases. We can use <img> and <script> tags that have the "src" attribute to extract images and external JavaScript files in the website. Similarly, CSS and anchor files are within "href" attribute in <link> and <a> tags. In Eqs. (1–4), basically we calculated the rate of img and script tags that have the “src” attribute, link and anchor tags that have “href” attribute to the total hyperlinks available in a webpage, these tags usually link to image, Javascript, anchor, and CSS files required for a website

1 $${\text{F}}3 = \left\{ {\begin{array}{*{20}l} {\frac{{{\text{F}}_{{{\text{Script}}\_{\text{files}}}} }}{{{\text{F}}_{{{\text{Total}}}} }}} & {if\;{\text{F}}_{{{\text{Total}}}} > 0} \\ 0 & {if\;{\text{F}}_{{{\text{Total}}}} = 0} \\ \end{array} } \right.$$

2 $${\text{F}}4 = \left\{ {\begin{array}{*{20}l} {\frac{{{\text{F}}_{{{\text{CSS}}\_{\text{files}}}} }}{{{\text{F}}_{{{\text{Total}}}} }}} & {if\;{\text{F}}_{{{\text{Total}}}} > 0} \\ 0 & {if\;{\text{F}}_{{{\text{Total}}}} = 0} \\ \end{array} } \right.$$

3 $${\text{F}}5 = \left\{ {\begin{array}{*{20}l} {\frac{{{\text{F}}_{{{\text{Img}}\_{\text{files}}}} }}{{{\text{F}}_{{{\text{Total}}}} }}} & {if\;{\text{F}}_{{{\text{Total}}}} > 0} \\ 0 & {if\;{\text{F}}_{{{\text{Total}}}} = 0} \\ \end{array} } \right.$$

4 $${\text{F}}6 = \left\{ {\begin{array}{*{20}l} {\frac{{{\text{F}}_{{{\text{a}}\_{\text{files}}}} }}{{{\text{F}}_{{{\text{Total}}}} }}} & {if\;{\text{F}}_{{{\text{Total}}}} > 0} \\ 0 & {if\;{\text{F}}_{{{\text{Total}}}} = 0} \\ \end{array} } \right.$$

where ${\text{F}}_{\text{Script}\_\text{files}}$, ${\text{F}}_{\text{CSS}\_\text{files}}$, ${\text{F}}_{\text{Img}\_\text{files}}$, ${\text{F}}_{\text{a}\_\text{files}}$ are the numbers of Javascript, CSS, image, anchor files existing in a webpage, and ${\text{F}}_{\text{Total}}$ is the total hyperlinks available in a webpage.

#### Empty hyperlinks (F7 and F8)

In the empty hyperlink, the “href” or “src” attributes of anchor, link, script, or img tags do not contain any URL. The empty link returns on the same webpage again when the user clicks on it. A benign website contains many webpages; thus, the scammer does not place any values in hyperlinks to make a phishing website behave like the benign website, and the hyperlinks look active on the phishing website. For example, <a href = “#”>, <a href = “#content”> and <a href = “javascript:void(0);”> HTML coding are used to design null hyperlinks ^24^. To establish the empty hyperlink features, we define the rate of empty hyperlinks to the total number of hyperlinks available in a webpage, and the rate of anchor tag without “href” attribute to the total number of hyperlinks in a webpage. Following formulas are used to compute empty hyperlink features

5 $$F7 = \left\{ {\begin{array}{*{20}l} {\frac{{{\text{F}}_{{{\text{a}}\_{\text{null}}}} }}{{{\text{F}}_{{{\text{Total}}}} }}} & {if\;{\text{F}}_{{{\text{Total}}}} > 0} \\ 0 & {if\;{\text{F}}_{{{\text{Total}}}} = 0} \\ \end{array} } \right.$$

6 $${\text{F}}8 = \left\{ {\begin{array}{*{20}c} {\frac{{{\text{F}}_{{{\text{Null}}}} }}{{{\text{F}}_{{{\text{Total}}}} }}} & {if\;{\text{F}}_{{{\text{Total}}}} > 0} \\ 0 & {if\;{\text{F}}_{{{\text{Total}}}} = 0} \\ \end{array} } \right.$$

where ${\text{F}}_{\text{a}\_\text{null}}$ and ${\text{F}}_{\text{null}}$ are the numbers of anchor tags without href attribute, and null hyperlinks in a webpage.

#### Total hyperlinks feature (F9)

Phishing websites usually contain minimal pages as compared to benign websites. Furthermore, sometimes the phishing webpage does not contain any hyperlink because the phishers usually only create a login page. Equation (7) computes the number of hyperlinks in a webpage by extracting the hyperlinks from an anchor, link, script, and img tags in the HTML source code.

7 $${\text{F}}9 = {\text{Total}}\;{\text{of}}\;{\text{hyperlinks}}\;{\text{present}}\;{\text{in}}\;{\text{a}}\;{\text{webpage}}$$

#### Internal and external hyperlinks (F10, F11, and F12)

The base domain name in the external hyperlink is different from the website domain name, unlike the internal hyperlink; the base domain name is the same as the website domain name. The phishing websites may contain many external hyperlinks that indicate to the target websites due to the cybercriminals commonly copy the HTML code from the targeted authorized websites to create their phishing websites. Most of hyperlinks in a benign website contain the similar base domain name, whereas many hyperlinks in a phishing site may include the corresponding benign website domain. In our approach, the internal and external hyperlinks are extracted from the “src” attribute of img, script, frame tags, “action” attribute of form tag, and “href” attribute of the anchor and link tags. We compute the rate of internal hyperlinks to the total links available in a webpage (Eq. 8) to establish the internal hyperlink feature, and the rate of external hyperlinks to the total links (Eq. 9) to set the external hyperlink feature. Moreover, to set the external/internal hyperlink feature, we compute the rate of external hyperlinks to the internal hyperlinks (Eq. 10). A specified number has been used as a way of detecting the suspected websites in some previous studies^5,9,24^ that these features used for classification. For example, if the rate of external hyperlinks to the total links is greater than 0.5, it will indicate that the website is phishing. However, determining a specific number as a parametric detection may cause errors in classification.

8 $${\text{F}}10 = \left\{ {\begin{array}{*{20}l} {\frac{{{\text{F}}_{{{\text{Internal}}}} }}{{{\text{F}}_{{{\text{Total}}}} }}} & {if\;{\text{F}}_{{{\text{Total}}}} > 0} \\ 0 & {if\;{\text{F}}_{{{\text{Total}}}} = 0} \\ \end{array} } \right.$$

9 $${\text{F}}11 = \left\{ {\begin{array}{*{20}l} {\frac{{{\text{F}}_{{{\text{External}}}} }}{{{\text{F}}_{{{\text{Total}}}} }}} & {if\;{\text{F}}_{{{\text{Total}}}} > 0} \\ 0 & {if\;{\text{F}}_{{{\text{Total}}}} = 0} \\ \end{array} } \right.$$

10 $${\text{F}}12 = \left\{ {\begin{array}{*{20}l} {\frac{{{\text{F}}_{{{\text{External}}}} }}{{{\text{F}}_{{{\text{Internal}}}} }}} & {if\;{\text{F}}_{{{\text{Internal}}}} > 0} \\ 0 & {if\;{\text{F}}_{{{\text{Internal}}}} = 0} \\ \end{array} } \right.$$

where ${\text{F}}_{\text{Internal}}$, ${\text{F}}_{\text{External}}$, and ${\text{F}}_{\text{Total}}$ are the number of external, internal, and total hyperlinks in a website.

#### Error in hyperlinks (F13)

Phishers sometimes add some hyperlinks in the fake website which are dead or broken links. In the hyperlink error feature, we check whether the hyperlink is a valid URL in the website. We do not consider the 403 and 404 error response code of hyperlinks due to the time consumed of the internet access to get the response code of each link. Hyperlink error is defined by dividing the total number of invalid links to the total links as represented in Eq. (11)

11 $${\text{F}}13 = \left\{ {\begin{array}{*{20}l} {\frac{{{\text{F}}_{{{\text{Error}}}} }}{{{\text{F}}_{{{\text{Total}}}} }}} & {if\;{\text{F}}_{{{\text{Total}}}} > 0} \\ 0 & {if\;{\text{F}}_{{{\text{Total}}}} = 0} \\ \end{array} } \right.$$

where ${\text{F}}_{\text{Error}}$ is the total invalid hyperlinks.

#### Login form features (F14 and F15)

In the fraudulent website, the common trick to acquire the user's personal information is to include a login form. In the benign webpage, the action attribute of login form commonly includes a hyperlink that has the similar base domain as appear in in the browser address bar^24^. However, in the phishing websites, the form action attribute includes a URL that has a different base domain (external link), empty link, or not valid URL (Eq. 13). The suspicious form feature (Eq. 14) is defined by dividing the total number of suspicious forms S to the total forms available in a webpage (Eq. 12)

12 $${\text{F}}14 = {\text{Total}}\;{\text{of}}\;{\text{forms}}\;{\text{present}}\;{\text{in}}\;{\text{a}}\;{\text{webpage}}$$

13 $${\text{S}} = \left\{ {\begin{array}{*{20}l} 1 \hfill & {if\;the\;URL\;of\;action\;field\;is\;Null} \hfill \\ 1 \hfill & {if\;the\;URL\;of\;action\;field\;is\;not\;valid} \hfill \\ 1 \hfill & {if\;the\;URL\;of\;action\;filed\;is\;external\;link} \hfill \\ 0 \hfill & {Otherwise} \hfill \\ \end{array} } \right.$$

14 $${\text{F}}15 = \left\{ {\begin{array}{*{20}c} {\frac{{{\text{F}}_{{\text{S}}} }}{{{\text{L}}_{{{\text{Total}}}} }}} & {if\;{\text{L}}_{{{\text{Total}}}} > 0} \\ 0 & {if\;{\text{L}}_{{{\text{Total}}}} = 0} \\ \end{array} } \right.$$

where ${\text{F}}_{\text{S}}$ and ${\text{L}}_{\text{Total}}$ are the number of suspicious forms and total forms present in a webpage.

**Figure 4:**
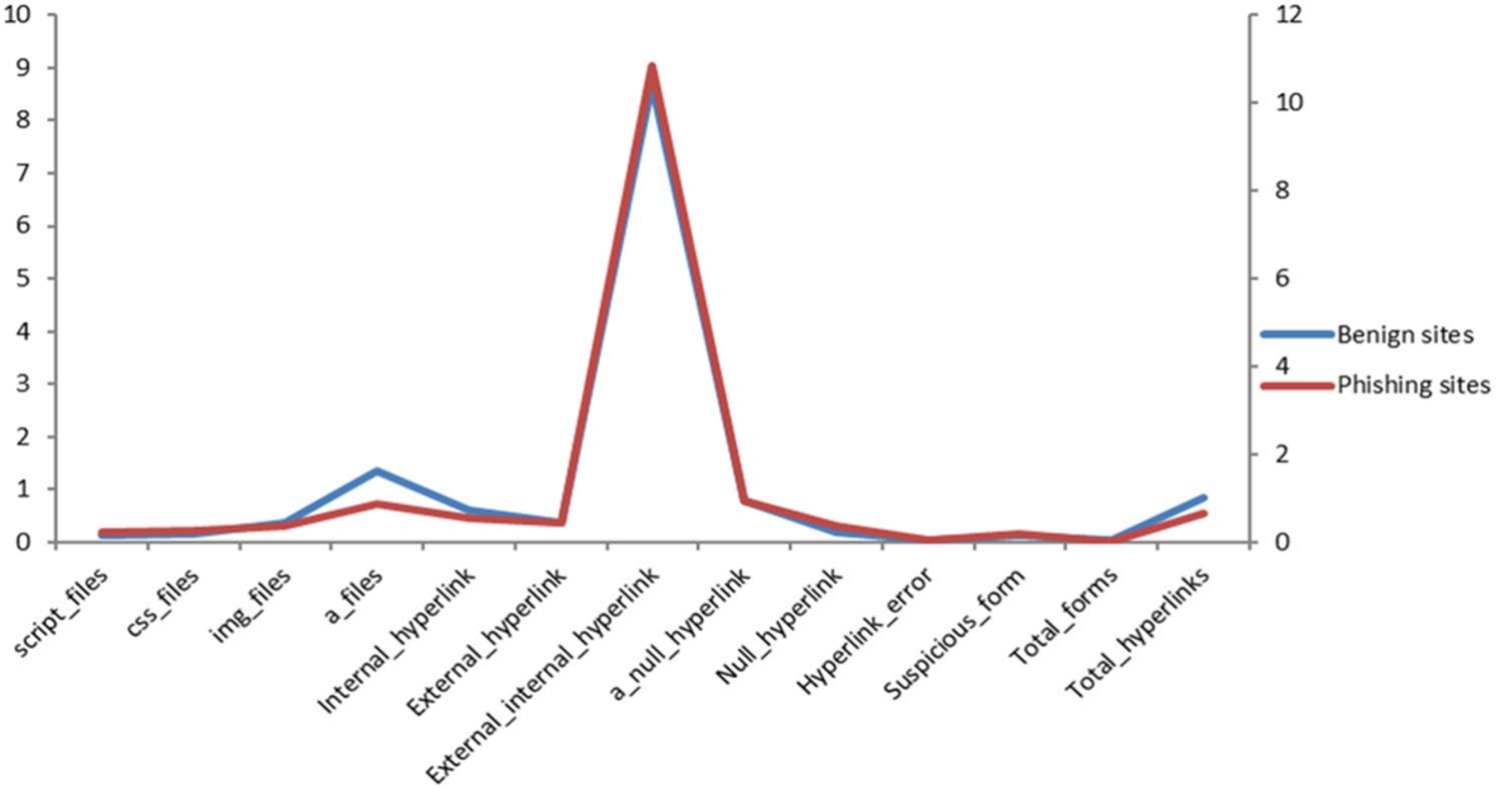
Distribution of hyperlink-based features in our data.

Figure 4 shows a comparison between benign and fishing hyperlink features based on the average occurrence rate per feature within each website in our dataset. From the figure, we noticed that the ratios of the external hyperlinks to the internal hyperlinks, and null hyperlinks in the phishing websites are higher than that in benign websites. Whereas, benign sites contain more anchor files, internal hyperlinks, and total hyperlinks.

### Classification algorithms

To measure the effectiveness of the proposed features, we have used various machine learning classifiers such as eXtreme Gradient Boosting (XGBoost), Random Forest, Logistic Regression, Naïve Bayes, and Ensemble of Random Forest and Adaboost classifiers to train our proposed approach. The major aim of comparing different classifiers is to expose the best classifier fit for our feature set. To apply different machine learning classifiers, Scikit-learn.org package is used, and Python is employed for feature extraction. From the empirical results, we noticed that XGBoost outperformed other classifiers. XGBoost algorithm is a type of ensemble classifiers, that transform weak learners to robust ones and convenient for our proposed feature set, thus it has high performance.

XGBoost (extreme gradient boosting) is a scalable machine learning system for tree boosting proposed by Chen and Guestrin^40^. Suppose there are $N$ websites in the dataset $\left\{ {\left( {x_{i} ,y_{i} } \right)|i = 1,2,...,N} \right\}$, where $x_{i} \in R^{d}$ is the extracted features associated with the $i - th$ website, $y_{i} \in \left\{ {0,\left. 1 \right\}} \right.$ is the class label, such that $y_{i} = 1$ if and only if the website is a labelled phishing website. The final output $f_{K} \left( x \right)$ of model is as follows^41,46^:

15 $$f_{k} \left( x \right)=l\left( {y_{i} ,f_{k} (x)} \right)=\sum\limits_{i = 1}^{N} {l\left( {y_{i} ,f_{k - 1} \left( {x_{i} } \right) + G_{k} \left( {x_{i} } \right)} \right)}+\Omega(G_{k})$$

where *l* is the training loss function and $\Omega \left( {G_{k}} \right) = \gamma T + \frac{1}{2}\lambda \sum\limits_{t = 1}^{T} {\omega_{t}^{2} }$ is the regulation term, since XGBoost introduces additive training and all previous *k-1* base learners are fixed, here we assumed that we are in step *k* that optimizes our function $f_{k} \left( x \right)$, *T* is the number of leaves nodes in the base learner *G*_*k*_, *γ* is the complexity of each leaf, λ is a parameter to scale the penalty, and *ω*_*t*_ is the output value at each final leaf node. If we apply the Taylor expansion to expand the Loss function at *f*_*k-1*_ (*x*) we will have^41^:

16 $$\begin{aligned} l\left( {y,f_{k - 1} \left( x \right) + G_{k} \left( x \right)} \right) & \approx \sum\limits_{i = 1}^{N} {l\left( {y_{i} ,f_{k - 1} \left( {x_{i} } \right) + G_{k} \left( {x_{i} } \right)} \right)} \\ & = \sum\limits_{i = 1}^{N} {\left( {l\left( {y_{i} ,f_{k - 1} \left( {x_{i} } \right)} \right) + g_{i} G_{k} \left( {x_{i} } \right) + \frac{1}{2}h_{i} G_{k}^{2} \left( {x_{i} } \right)} \right)} + \gamma T + \frac{1}{2}\lambda \sum\limits_{t = 1}^{T} {\omega_{t}^{2} } \\ \end{aligned}$$

where $g_{i} = \frac{{\partial l\left( {y_{i} ,f_{k - 1} \left( {x_{i} } \right)} \right)}}{{\partial f_{k - 1} \left( x \right)}},h_{i} = \frac{{\partial l\left( {y_{i} ,f_{k - 1} \left( {x_{i} } \right)} \right)}}{{\partial f_{k - 1}^{2} \left( x \right)}}$ are respectively first and second derivative of the Loss function.

XGBoost classifier is a type of ensemble classifiers, that transform weak learners to robust ones and convenient for our proposed feature set for the prediction of phishing websites, thus it has high performance. Moreover, XGBoost provides a number of advantages, some of which include: (i) The strength to handle missing values existing within the training set, (ii) handling huge datasets that do not fit into memory and (iii) For faster computing, XGBoost can make use of multiple cores on the CPU. The websites are classified into two possible categories: phishing and benign using a binary classifier. When a user requests a new site, the trained XGBoost classifier determines the validity of a particular webpage from the created feature vector.

## Experiments and result analysis

In this section we describe the training and testing dataset, performance metrics, implementation details, and outcomes of our approach. The proposed features described in “Features extraction” section are used to build a binary classifier, which classify phishing and benign websites accurately.

### Dataset

We collected the dataset from two sources for our experimental implementation. The benign webpages are collected in February 2020 from Stuff Gate^42^, whereas the phishing webpages are collected from PhishTank^43^, which have been validated from August 2016 to April 2020. Our dataset consists of 60,252 webpages and their HTML source codes, wherein 27,280 ones are phishing and 32,972 ones are benign. Table 3 provides the distribution of the benign and phishing instances. We have divided the dataset into two groups where D1 is our dataset, and D2 is dataset used in existing literature^6^. The database management system (i.e., pgAdmin) has been employed with python to import and pre-process the data. The data sets were randomly split in 80:20 ratios for training and testing, respectively.

**Table 3:** Data distribution.

Dataset	Benign webpages	Phishing webpages	Total
D1	32,972	27,280	60,252
D2	36,400	37,175	73,575

### Performance metrics

To measure the performance of proposed anti-phishing approach, we used different statistical metrics such true-positive rate (TPR), true-negative rate (TNR), false-positive rate (FPR), false-negative rate (FNR), sensitivity or recall, accuracy (Acc), precision (Pre), F-Score, AUC, and they are presented in Table 4. ${N}_{B}$ and ${N}_{P}$ indicate the total number of benign and phishing websites, respectively. ${N}_{B\to B}$ are the benign websites are correctly marked as benign, ${N}_{B\to P}$ are the benign websites are incorrectly marked as phishing, ${N}_{P\to P}$ are the phishing websites are correctly marked as phishing, and ${N}_{P\to B}$ are the phishing websites are incorrectly marked as benign. The receiver operating characteristic (ROC) arch and AUC are commonly used to evaluate the measures of a binary classifier. The horizontal coordinate of the ROC arch is FPR, which indicates the probability that the benign website is misclassified as a phishing; the ordinate is TPR, which indicates the probability that the phishing website is identified as a phishing.

**Table 4:** Different statistics metrics used to measure the performance of our approach.

Formula	Description
$TPR = \frac{{N_{p \to p} }}{{N_{p} }} \times 100$	The ratio of phishing websites classified as phishing to the total number of phishing websites
$TNR = \frac{{N_{B \to B} }}{{N_{B} }} \times 100$	The ratio of benign websites classified as benign to the total number of benign websites
$FPR = \frac{{N_{B \to p} }}{{N_{B} }} \times 100$	The ratio of benign websites classified as phishing to the total number of benign websites
$FNR = \frac{{N_{p \to B} }}{{N_{p} }} \times 100$	The ratio of phishing websites classified as benign to the total number of phishing websites
$Accuracy = \frac{{\left( {TP + TN} \right)}}{{\left( {TP + TN + FP + FN} \right)}} \times 100$	The ratio of phishing and benign websites which are correctly classified to the total number of all websites
$Precision = \frac{{\left( {TP} \right)}}{{\left( {TP + FP} \right)}} \times 100$	The ratio of phishing websites which are correctly classified as phishing to the total number of phishing websites classified as phishing and benign websites classified as phishing
$Recall = \frac{{\left( {TP} \right)}}{{\left( {TP + FN} \right)}} \times 100$	The ratio of phishing websites which are correctly classified to the total number of all phishing websites
$F - Score = \frac{{\left( {2 \times precision \times recall} \right)}}{{\left( {precision + recall} \right)}} \times 100$	A weighted harmonic average of precision and recall rate

### Evaluation of features

**Figure 5:**
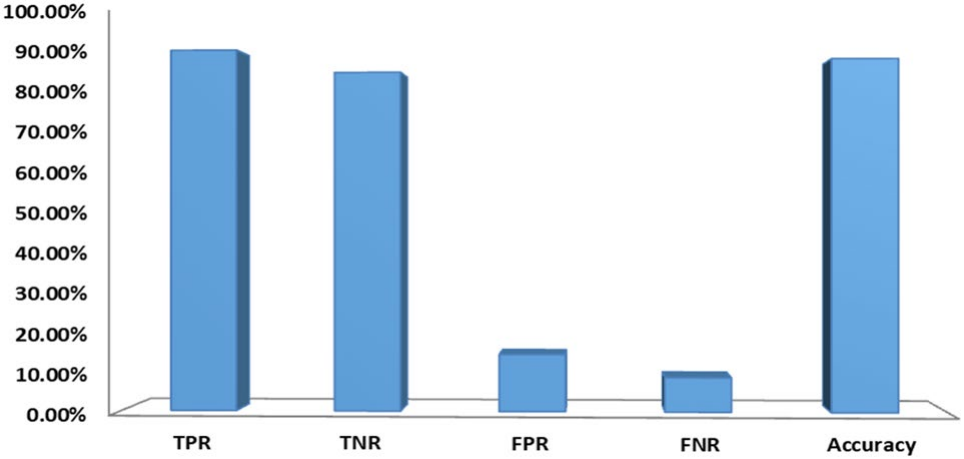
Performance of textual content features.

In this section, we evaluated the performance of our proposed features (URL and HTML). We have implemented different Machine Learning (ML) classifiers for feature evaluation used in our approach. In Table 5, we extracted various text features such as TF-IDF word level, TF-IDF N-gram level (the length of n-gram between 2 and 3), TF-IDF character level, count vectors (bag-of-words), word sequences vectors, global to vector (GloVe) pre-trained word embedding, trained word embedding, character sequences vectors and implemented various classifiers such as XGBoost, Random forest, logistic regression, Naïve Bayes, Deep Neural Networks (DNN), Convolutional Neural Networks (CNN), and Long Short-Term Memory (LSTM) network. The main intention of this experiment was to reveal the best textual content features convenient for our data. From the experimental results, it is noticed that TF-IDF character level features outperformed other features with significant accuracy, precision, F-Score, Recall, and AUC using XGBoost and DNN classifiers. Hence, we implemented TF-IDF character level technique to generate text features (F2) of the webpage. Figure 5 presents the performance of textual content-based features. As shown in the figure, text features can correctly filter a high amount of phishing websites and achieved an accuracy of 88.82%.

**Table 5:** Performance of different textual based features on dataset D1 with various classifiers.

Classifier	Textual content features	Pre (%)	Recall (%)	F-Score (%)	AUC (%)	Acc (%)
LR	TF-IDF word level	85.68	88.25	86.95	85.38	85.62
	TF-IDF N-gram level	85.23	85.42	85.33	83.93	84.05
	TF-IDF character level	84.55	87.15	85.83	84.13	84.39
	Count vectors	86.84	79.12	82.80	82.45	82.16
	Word sequences vectors	55.87	83.27	66.87	52.61	55.23
XGBoost	TF-IDF word level	88.44	88.56	88.50	87.41	87.52
	TF-IDF N-gram level	87.77	86.51	87.13	86.10	86.14
	TF-IDF character level	**89.01**	**90.58**	**89.79**	**88.65**	**88.82**
	Word sequences vectors	82.66	85.87	84.23	82.24	82.55
	Count vectors	88.26	87.75	88.00	86.95	87.02
	Character sequences vectors	81.47	87.81	84.52	82.05	82.54
RF	TF-IDF word level	85.94	92.67	89.18	87.34	87.80
	TF-IDF N-gram level	86.77	89.57	88.14	86.68	86.93
	TF-IDF character level	85.44	92.81	88.97	87.02	87.51
	Count vectors	85.81	93.08	89.30	87.41	87.90
	Word sequences vectors	81.56	90.71	85.89	83.19	83.83
	Character sequences vectors	79.51	93.91	86.11	82.60	83.56
NB	TF-IDF word level	84.50	79.12	81.72	80.95	80.79
	TF-IDF N-gram level	82.45	71.16	76.39	76.59	76.13
	TF-IDF character level	76.45	81.89	79.08	75.98	76.49
	Count vectors	82.62	71.63	76.74	76.88	76.43
	Word sequences vectors	62.89	42.66	50.83	56.39	55.22
DNN	TF-IDF word level	87.08	91.20	89.09	87.57	87.88
	TF-IDF N-gram level	88.12	84.29	86.17	85.40	85.31
	TF-IDF character level	**88.32**	**91.62**	**89.94**	**88.62**	**88.40**
	Count vectors	87.49	89.46	88.47	87.14	87.34
	Word sequences vectors	54.26	100.0	70.35	50.0	54.26
	Character sequences vectors	76.41	91.43	83.25	78.97	80.03
LSTM	GloVe pre-trained word embedding	87.05	90.79	88.88	87.38	87.67
	Trained word embedding	88.14	89.20	88.66	87.48	87.62
CNN	Character embedding	82.06	89.34	85.54	83.08	83.61
	Trained word embedding	89.57	85.00	87.22	86.62	86.49

Significant values are in [bold].

**Figure 6:**
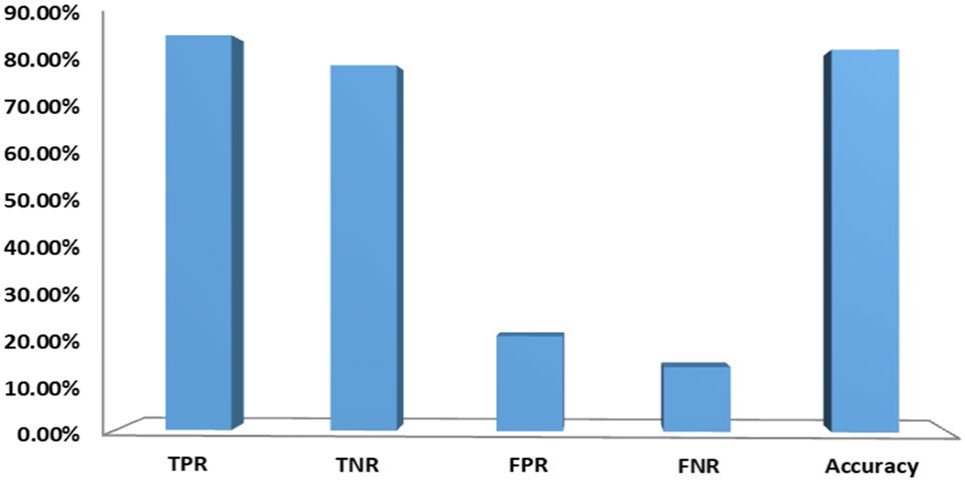
Performance of hyperlink based features.

Table 6 shows the experiment results with hyperlinks features. From the empirical results, it is noticed that Random Forest classifier superior to the other classifiers with an accuracy of 82.27%, precision of 77.59%, F_Measure of 81.63%, recall of 86.10%, and AUC of 82.57%. It is also noticed that ensemble and XGBoost classifiers attained good accuracy of 82.18% and 80.49%, respectively. Figure 6 presents the classification results of hyperlink based features (F3–F15). As shown in the figure, hyperlink based features can accurately clarify 79.04% of benign websites and 86.10% of phishing websites.

**Table 6:** Performance of the proposed hyperlink features on D1 with various classifiers.

Classifier	Precision (%)	Recall (%)	F_Measure (%)	AUC (%)	Accuracy (%)
RF	**77.59**	**86.10**	**81.63**	**82.57**	**82.27**
Ensemble	77.39	86.23	81.57	82.50	82.18
LR	69.05	55.67	61.65	67.32	68.31
NB	68.31	31.60	43.21	59.62	62.01
XGBoost	75.55	84.77	79.90	80.82	80.49

Significant values are in [bold].

**Figure 7:**
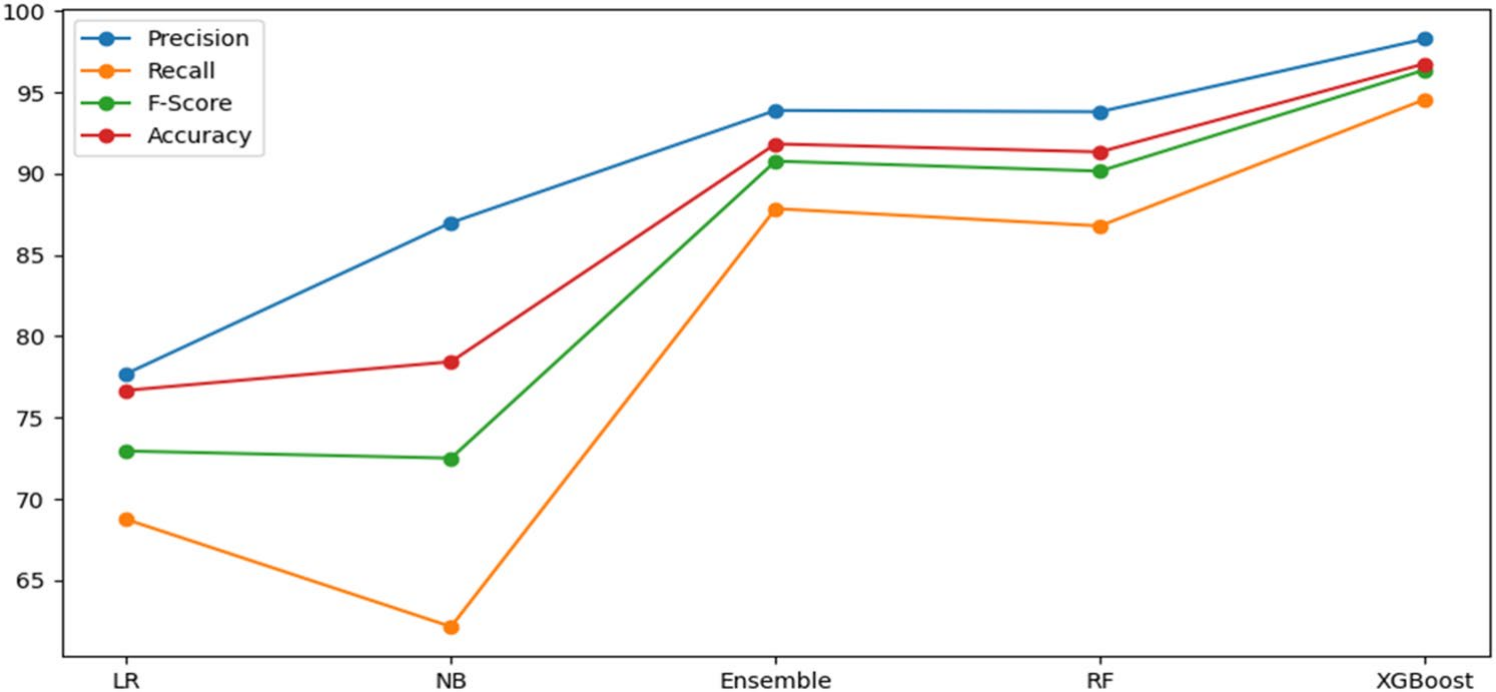
Test results of various classifiers with respect to combined features.

**Figure 8:**
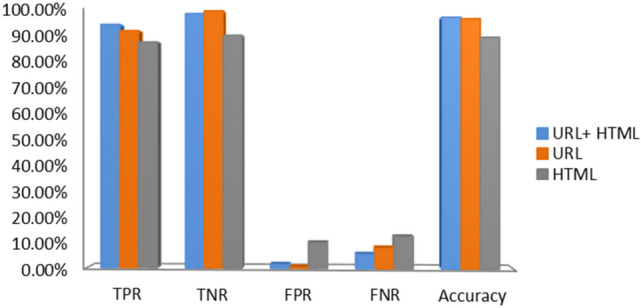
Performance of different feature combinations using XGBoost on dataset D1.

In Table 7, we integrated features of URL and HTML (hyperlink and text) using various classifiers to verify complementary behavior in phishing websites detection. From the empirical results, it is noticed that LR classifier has sufficient accuracy, precision, F-Score, AUC, and recall in terms of the HTML features. In contrast, NB classifier has good accuracy, precision, F-Score, AUC, and recall with respect to combining all the features. RF and ensemble classifiers achieved high accuracy, recall, F-Score, and AUC with respect to URL based features. XGBoost classifier outperformed the others with an accuracy of 96.76%, F-Score of 96.38%, AUC of 96.58% and recall of 94.56% with respect to combining all the features. It is observed that URL and HTML features are valuable in phishing detection. However, one type of feature is not suitable to identify all kinds of phishing webpages and does not result in high accuracy. Thus, we have combined all features to get more comprehensive features. The results on various classifiers of combined feature set are also shown in Fig. 7. In Fig. 8 we compare the three feature sets in terms of accuracy, TNR, FPR, FNR, and TPR.

**Table 7:** Performance of different feature combinations on dataset D1 with various classifiers.

Classifier	Features	Pre (%)	Recall (%)	F-Score (%)	AUC (%)	ACC (%)
LR	F_URL_	74.67	67.92	71.13	74.25	74.79
	F_HTML_	83.50	81.98	82.74	84.16	84.35
	F_URL+ HTML_	77.71	68.74	72.95	76.06	76.68
NB	F_URL_	81.41	22.09	34.76	58.92	62.06
	F_HTML_	65.67	87.57	75.06	74.49	73.38
	F_HTM + URL_	86.99	62.15	72.51	77.16	78.44
Ensemble	F_URL_	98.42	92.05	95.13	95.40	95.69
	F_HTML_	90.22	82.01	85.92	87.25	87.70
	F_URL+ HTML_	93.89	87.85	90.77	91.52	91.83
RF	F_URL_	98.54	92.14	95.23	95.49	95.78
	F_HTML_	90.77	81.98	86.16	87.48	87.95
	F_URL + HTML_	93.81	86.79	90.16	90.98	91.34
XGBoost	F_URL_	99.58	92.27	95.79	95.97	96.29
	F_HTML_	88.21	87.68	87.94	88.90	89.01
	F_URL + HTML_	**98.28**	**94.56**	**96.38**	**96.58**	**96.76**

Significant values are in [bold].

**Figure 9:**
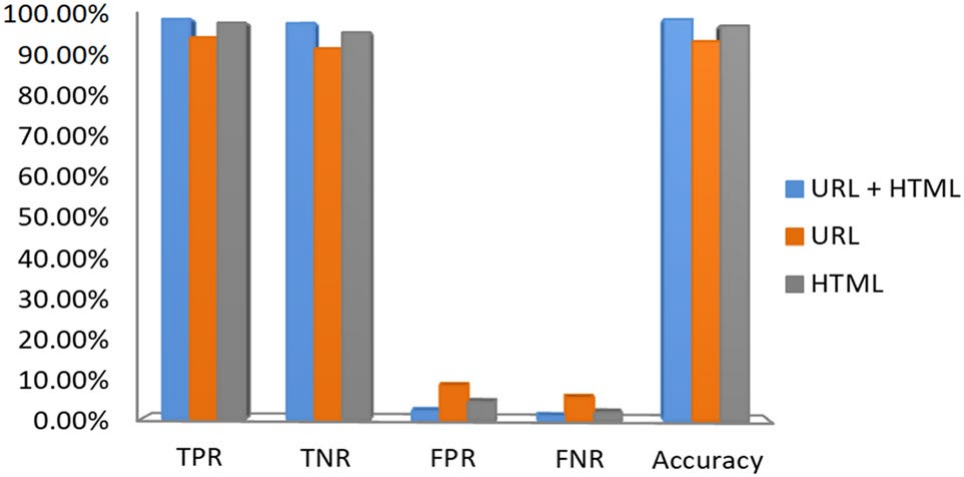
Performance of the proposed approach on dataset D2.

The confusion matrix is used to measure results where each row of the matrix represents the instances in a predicted class, while each column represents the instances in an actual class (or vice versa). The confusion matrix of the proposed approach is created as represented in Table 8. From the results, combining all kind of features together as an entity correctly identified 5212 out of 5512 phishing webpages and 6448 out of 6539 benign webpages and attained an accuracy of 96.76%. Our approach results in low false positive rate (i.e., less than 1.39% of benign webpages incorrectly classified as phishing), and high true positive rate (i.e., more than 94.56% of phishing webpages accurately classified). We have also tested our feature sets (URL and HTML) on the existing dataset D2. Since dataset D2 only contains legitimate and malicious URLs, we needed to extract the HTML source code features for these URLs. The results are given in Table 9 and Fig. 9. From the results, it is noticed that combining all kinds of features had outperformed other feature sets with a significant accuracy of 98.48%, TPR of 99.04%, and FPR of 2.09%.

**Table 8:** Confusion matrix of the proposed approach on dataset D1.

Confusion matrix	Predicted	
	P	N
**URL based features**		
Actual		
P	5086	426
N	21	6518
**HTML based features**		
Actual		
P	4833	679
N	646	5893
**URL + HTML based features**		
Actual		
P	5212	300
N	91	6448

**Table 9:** Results of the proposed approach on dataset D2.

Features	Pre (%)	Recall (%)	F-Score (%)	AUC (%)	Acc (%)
F_URL_	92.16	94.58	93.36	93.11	93.14
F_HTML_	95.84	98.05	96.94	96.82	96.84
F_URL + HTML_	**98.01**	**99.04**	**98.52**	**98.47**	**98.48**

Significant values are in [bold].

### Comparison with existing approaches

In this experiment, we compare our approach with existing anti-phishing approaches. Notice that we have applied Le et al.^29^ and Aljofey et al.^3^ works on dataset D1 to evaluate the efficiency of the proposed approach. While for comparison of the proposed approach with Sahingoz et al.^6^, Rao et al.^13^, Chatterjee and Namin^30^ works, we evaluated our approach on benchmark dataset D2^6,13,30^ based on the four-statistics metrics used in the papers. The comparison results are shown in Table 10. From the results, it is observed that our approach gives better performance than other approaches discussed in the literature, which shows the efficiency of detecting phishing websites over the existing approaches.

**Table 10:** Comparison of the proposed approach with other standard approaches on data set D2.

Author	Pre (%)	Recall (%)	F-Score (%)	Acc (%)
Sahingoz et al.^6^	97.00	99.00	98.00	97.98
Rao et al.^13^	98.04	98.42	98.23	98.25
Chatterjee and Namin^30^	86.71	88.00	87.30	90.10
Proposed approach	**98.01**	**99.04**	**98.52**	**98.48**

Significant values are in [bold].

In Table 11, we implemented Le et al.^29^ and Aljofey et al.^3^ methods to our dataset D1 and our approach outperformed the others with an accuracy of 96.76%, precision of 98.28%, and F-Score of 96.38%. It should also be mentioned that Aljofey et al. method achieved 97.86% recall, which is 3.3% greater than our method, whereas our approach gives TNR that is higher by 4.97%, and FPR that is lesser by 4.96%. Our approach accurately identifies the legitimate websites with a high TNR and low FPR. Some phishing detection methods achieve high recall, however inaccurate classification of the legitimate websites is more serious compared to inaccurate classification of the phishing sites.

**Table 11:** Comparison of the proposed approach with other standard approaches on dataset D1.

Author	Pre (%)	Recall (%)	F-Score (%)	Acc (%)	TNR (%)	FPR (%)
Le et al.^29^	96.38	90.06	93.12	93.91	97.15	2.84
Aljofey et al.^3^	94.84	97.86	96.33	95.94	93.64	6.35
Proposed approach	**98.28**	**94.56**	**96.38**	**96.76**	**98.61**	**1.39**

Significant values are in [bold].

## Discussion and limitations

The phishing website seems similar to its benign official website, and the defiance is how to distinguish between them. This paper proposed a novel anti-phishing approach, which involves different features (URL, hyperlink, and text) that have never been taken into consideration. The proposed approach is a completely client-side solution. We applied these features on various machine learning algorithms and found that XGBoost attained the best performance. Our major aim is to design a real-time approach, which has a high true-negative rate and low false-positive rate. The results show that our approach correctly filtered the benign webpages with a low amount of benign webpages incorrectly classified as phishing. In the process of phishing webpage classification, we construct the dataset by extracting the relevant and useful features from benign and phishing webpages.

A desktop machine having a core™ i7 processor with 3.4 GHz clock speed and 16 GB RAM is used to executed the proposed anti-phishing approach. Since Python provides excellent support of its libraries and has sensible compile-time, the proposed approach is implemented using Python programming language. BeautifulSoup library is employed to parse the HTML of the specified URL. The detection time is the time between entering URL to generating outputs. When the URL is entered as a parameter, the approach attempts to fetch all specific features from the URL and HTML code of the webpage as debated in feature extraction section. This is followed by current URL classification in form of benign or phishing based on the value of the extracted feature. The total execution time of our approach in phishing webpage detection is around 2–3 s, which is quite low and acceptable in a real-time environment. Response time depends on different factors, such as input size, internet speed, and server configuration. Using our data D1, we also attempted to compute the time taken for training, testing and detecting of proposed approach (all feature combinations) for the webpage classification. The results are given in Table 12.

**Table 12:** Training, testing and detection time of the proposed approach on D1.

Features	Training time (s)	Test time (s)	Detection time (s)
F_URL_	12.7969	0.0548	0.356995
F_HTML_	1467.12	23.328	26,380.12
F_URL + HTML_	9352.64	33.338	26,371.05

**Figure 10:**
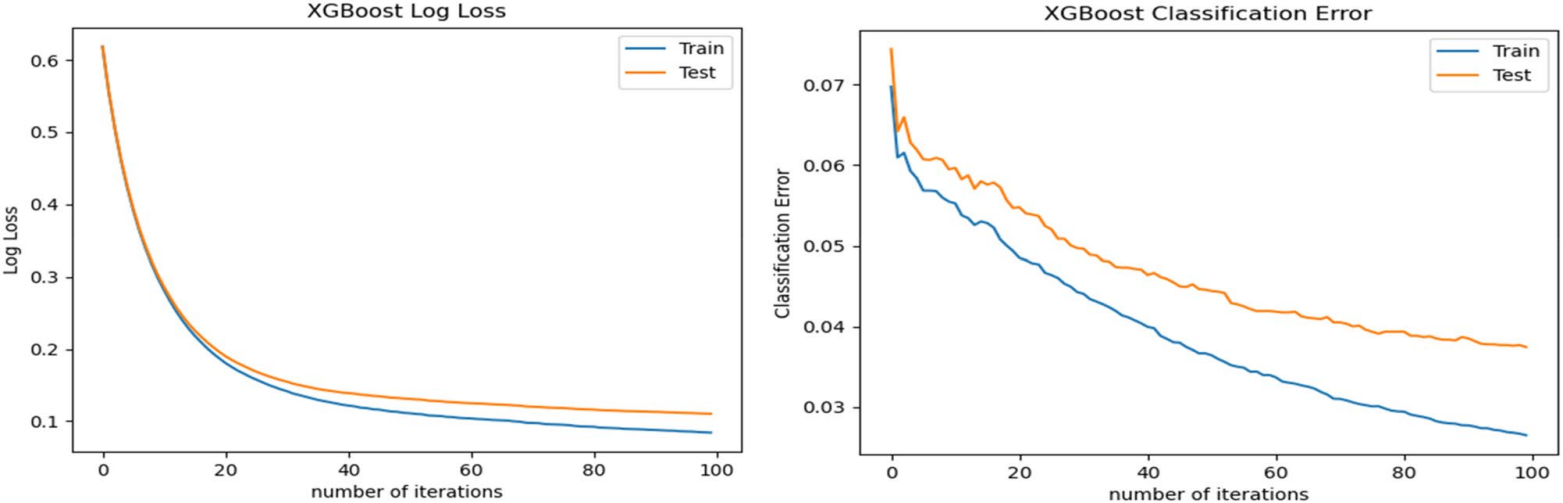
XGBoost learning curve of logarithmic loss and classification error on dataset D1.

In pursuit of a further understanding of the learning capabilities, we also present the classification error as well as log loss regarding the number of iterations implemented by XGBoost. Log loss, short for logarithmic loss is a loss function for classification that indicates the price paid for the inaccuracy of predictions in classification problems. Figure 10 show the logarithmic loss and the classification error of the XGBoost approach for each epoch on the training and test dataset D1. From reviewing the figure, we might note that the learning algorithm is converging after approximately 100 iterations.

### Limitations

Although our proposed approach has attained outstanding accuracy, it has some limitations. First limitation is that the textual features of our phishing detection approach depend on the English language. This may cause an error in generating efficient classification results when the suspicious webpage includes language other than English. About half (60.5%) of the websites use English as a text language^44^. However, our approach employs URL, noisy part of HTML, and hyperlink based features, which are language-independent features. The second limitation is that despite the proposed approach uses URL based features, our approach may fail to identify the phishing websites in case when the phishers use the embedded objects (i.e., Javascript, images, Flash, etc.) to obscure the textual content and HTML coding from the anti-phishing solutions. Many attackers use single server-side scripting to hide the HTML source code. Based on our experiments, we noticed that legitimate pages usually contain rich textual content features, and high amount of hyperlinks (At least one hyperlink in the HTML source code). At present, some phishing webpages include malware, for example, a Trojan horse that installs on user’s system when the user opens the website. Hence, the next limitation of this approach is that it is not sufficiently capable of detecting attached malware because our approach does not read and process content from the web page's external files, whether they are cross-domain or not. Finally, our approach's training time is relatively long due to the high dimensional vector generated by textual content features. However, the trained approach is much better than the existing baseline methods in terms of accuracy.

## Conclusion and future work

Phishing website attacks are a massive challenge for researchers, and they continue to show a rising trend in recent years. Blacklist/whitelist techniques are the traditional way to alleviate such threats. However, these methods fail to detect non-blacklisted phishing websites (i.e., 0-day attacks). As an improvement, machine learning techniques are being used to increase detection efficiency and reduce the misclassification ratio. However, some of them extract features from third-party services, search engines, website traffic, etc., which are complicated and difficult to access. In this paper, we propose a machine learning-based approach which can speedily and precisely detect phishing websites using URL and HTML features of the given webpage. The proposed approach is a completely client-side solution, and does not rely on any third-party services. It uses URL character sequence features without expert intervention, and hyperlink specific features that determine the relationship between the content and the URL of a webpage. Moreover, our approach extracts TF-IDF character level features from the plaintext and noisy part of the given webpage's HTML.

A new dataset is constructed to measure the performance of the phishing detection approach, and various classification algorithms are employed. Furthermore, the performance of each category of the proposed feature set is also evaluated. According to the empirical and comparison results from the implemented classification algorithms, the XGBoost classifier with integration of all kinds of features provides the best performance. It acquired 1.39% false-positive rate and 96.76% of overall detection accuracy on our dataset. An accuracy of 98.48% with a 2.09% false-positive rate on a benchmark dataset.

In future work, we plane to include some new features to detect the phishing websites that contain malware. As we said in “Limitations” section, our approach could not detect the attached malware with phishing webpage. Nowadays, blockchain technology is more popular and seems to be a perfect target for phishing attacks like phishing scams on the blockchain. Blockchain is an open and distributed ledger that can effectively register transactions between receiving and sending parties, demonstrably and constantly, making it common among investors^45^. Thus, detecting phishing scams in the blockchain environment is a defiance for more research and evolution. Moreover, detecting phishing attacks in mobile devices is another important topic in this area due to the popularity of smart phones^47^, which has made them a common target of phishing offenses.

## Data Availability

The dataset generated during the current study are available in the Google Drive repository: https://drive.google.com/file/d/18ZZHsCeMmF9HKTaL_yd41oJ_3Fgk0gWE/view?usp=sharing.
